# Molecular Characterization of Major Structural Protein Genes of Avian Coronavirus Infectious Bronchitis Virus Isolates in Southern China

**DOI:** 10.3390/v5123007

**Published:** 2013-12-04

**Authors:** Mei-Lan Mo, Meng Li, Bai-Cheng Huang, Wen-Sheng Fan, Ping Wei, Tian-Chao Wei, Qiu-Ying Cheng, Zheng-Ji Wei, Ya-Hui Lang

**Affiliations:** 1 College of Animal Science and Technology, Guangxi University, 100 Daxue Road, Nanning, Guangxi 530004, China; E-Mails: mengli4836@163.com(M.L.); hbch228@163.com (B.-C.H.); 2003424318@163.com (W.-S.F.); tcwei88@126.com (T.-C.W.); zhengjiwei @sohu.com (Z.-J.W.); langyahui@163.com (Y.-H.L.); 2 Yuyao Poultry and Livestock Disease Prevention and Cure Institute, 189 Fengshan Road, Yuyao, Zhejiang 315400, China; E-Mail: gxdxchenqy@126.com (Q.-Y.C.)

**Keywords:** infectious bronchitis virus, genetic variation, phylogenetic tree, entropy, positive selection, recombination

## Abstract

To gain comprehensive genetic information of circulating avian coronavirus infectious bronchitis virus (IBV) isolates in China, analysis of the phylogenetic tree, entropy of the amino acid sequences, and the positive selection as well as computational recombinations of S1, M and N genes of 23 IBV isolates was conducted in the present study. The phylogenetic trees based on the S1, M and N genes exhibited considerably different topology and the CK/CH/LSC/99I-type isolates were the predominant IBVs based on the phylogenetic analysis of S1 gene. Results of entropy of amino acid sequences revealed that the S1 gene had the largest variation; the M gene had less variation than the N gene. Positive selections were detected in not only S1 but also M and N gene proteins. In addition, five S1 gene recombinants between vaccine strain 4/91 and CK/CH/LSC/99I-type field isolate were confirmed. In conclusion, multiple IBV genotypes co-circulated; genetic diversity and positive selections existed in S1, M and N genes; 4/91 vaccine recombinants emerged in China. Our results show that field IBVs in China are continuing to evolve and vaccine strains may have an important role in the appearance of new IBV strains via recombination. In addition, the present study indicates that IBV evolution is driven by both generations of genetic diversity and selection.

## 1. Introduction

Infectious bronchitis (IB) is an acute, highly infectious and contagious disease of domestic chickens worldwide caused by avian infectious bronchitis virus (IBV), a member of genus *Gammacoronavirus*, subfamily *Coronavirinae,* family *Coronaviridae* [1]. IB affects chickens of all ages and IBV replicates primarily in the respiratory tract, and also in some epithelial cells of the kidney, gut and oviduct, resulting in reduced performance, reduced egg quality and quantity, increased susceptibility to infections with other pathogens, and condemnations at processing [2]. Multiple IBV serotypes or genotypes have been identified worldwide and different serotypes of IBVs confer little or no cross-protection against the others. 

IBV genome consists of a linear, single-stranded, positive-sense RNA of 27.6 kb, which encodes four major structural proteins, the spike (S) glycoprotein, the membrane (M) glycoprotein, the nucleocapsid (N) protein and the envelope or small membrane (E) protein [3]. The S glycoprotein is post-translationally cleaved into S1 and S2 subunits and S1 is the most divergent region, which carries conformationally-dependent virus-neutralizing and serotype-specific epitopes [4,5]. The N protein located in the capsid of the virion is involved in RNA replication, assembly and carries group-specific antigenic determinants [6] and has high immunogenicity, readily inducing antibodies and cytotoxic T-lymphocyte immunity in chickens [7]. S1 and N genes have been used most frequently to determine the relatedness of emerging strains of IBV [5,8]. The M protein is a structural membrane protein and plays an important role in the viral assembly process and particularly is indispensable for many biological functions including viral core stability. Interactions of M and E proteins are important for virus budding and formation of virus-like particles, which are involved in mucosal immunity [9]. 

The genetic diversity and viral evolution of IBV are mainly monitored by analysis of the S1 gene because of its high variability and close serotype correlation [10], but viruses within the same serotype can have a high degree of genetic variability outside of the spike gene [11]. Pathogenicity of IBV is associated with the spike gene as well as genes outside of the spike gene [12]. The M protein is associated with virus assembly and change this protein will affect the efficiency of virus particles formation and subsequent transmission of the virus [3]. The N protein plays an important role in viral replication, assembly, and immunity. In addition to S1 glycoprotein, the N protein could represent an important target in the prevention of IB outbreaks [13]. Recent evidence revealed that there are significant variations in the N and M genes between strains [13,14]. Therefore, it is necessary to analyze multiple genes especially to analyze the genetic variation of S1, M and N genes considering their importance as structural proteins. 

The major challenge for the prevention and control of IB is the increasing number of new serotypes or variants of IBV, which was caused by frequent gene mutation and recombination [15,16,17,18]. Recombination is thought to be a contributing factor in the emergence and evolution of IBV or even the emergence of new coronaviruses and new diseases [3]. The studies of IBV recombination are very important for IBV control, because they will further our understanding of the diversity and evolution mechanisms of these viruses and thus enable the development of better control methods [3,18].

IBV strains within a geographic region are unique and distinct [19] although many countries share some common antigenic types. Therefore, it is extremely critical to identify the prevalence of IBVs and genetic characteristics of circulating strains in a region or a country in order to develop effective vaccines for the control of the disease. Outbreaks of IB have been occurring frequently in China in spite of intensive vaccinations for many years [15,17,20,21,22]. IB is still a major problem in Guangxi province [15,17], which is located in southern China and produces a total of 700 million birds per year and ranks third in China [23]. It is very important to know the genetic characteristics of prevalent strains of IBVs in this region. We previously reported the genotype diversity of Guangxi IBV isolates based on the hypervariable region I (HVR I) of S1 gene [15], but the available comprehensive genetic information of circulating IBV strains in this region was limited. Therefore, in the present study we performed the analysis of the phylogenetic tree, of the entropy of the amino acid sequences, of positive selection as well as of computational recombination based on the sequencing results of the viral structural protein genes S1, M and N in order to provide molecular epidemiology information of IBV and to lay a good foundation for the control of IB in the field.

## 2. Results

### 2.1. Alignment Analysis of Nucleotide and the Deduced Amino Acid Sequences

The nucleotide and deduced amino acid (aa) sequence identities of the S1, M and N genes among the 23 isolates were 76.1%–99.9% (aa: 74.7%–99.8%), 88.1%–100.0% (aa: 89.1%–100.0%) and 86%–100.0% (aa: 90.4%–100.0%), respectively. The identities of nucleotide and deduced amino acid sequences of S1, M and N genes between the 23 isolates and all the reference strains were 57.9%–99.9% (aa: 47.2%–99.8%), 78.5%–100.0% (aa: 66.2%–100.0%) and 85.6%–99.9% (aa: 83.4%–99.7%), respectively. Compared with the most popularly used vaccine strain H120, all the isolates had lower nucleotide sequence identities (S1: 76.2%–83%; M: 87.9%–90.2%; N: 85.7%–87.9%) except for GX-NN1 and GX-NN2 (99.5% and 99.7) in the S1 gene, GX-NN1, GX-NN2 and GX-NN5 (99.8%, 99.7% and 99.8%) in the M gene, GX-NN5 (99.1%) in the N gene (Supplementary Table S1). 

### 2.2. Phylogenetic Analysis

**Figure 1:**
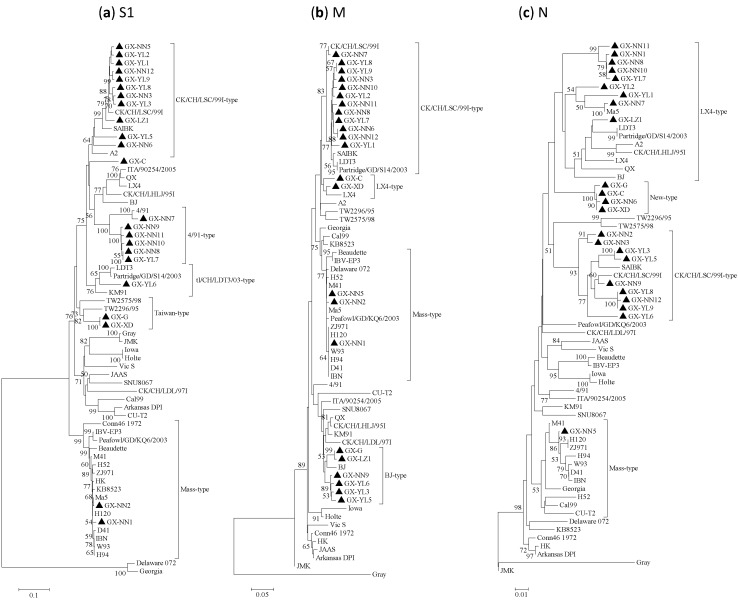
Phylogenetic trees of gene (**a**) S1, (**b**) M and (**c**) N of infectious bronchitis viruses (IBVs), where the 23 IBV strains are marked with filled triangle. Phylogenetic trees were constructed with the neighbor-joining method using MEGA 5.05 version. The bootstrap values were determined from 1000 replicates of the original data. The branch number represents the percentage of times that the branch appeared in the tree. Bootstrap values greater than 70% are shown. The p-distance is indicated by the bar at the bottom of the figure.

Both the phylogenetic trees constructed with the Neighbor-joining and Maximum-likelihood method had very similar topography, so only the Neighbor-joining trees are shown (Figure 1). The phylogenetic trees based on S1 gene amino acid sequences showed that all IBV isolates except GX-C were divided into five distinct groups (Figure 1a). Eleven out of 23 isolates were grouped into the CK/CH/LSC/99I-type with China IBV reference strains CK/CH/LSC/99I, SAIBK and A2, which were isolated during 2004–2008. Isolates GX-NN7, GX-NN11, GX-NN9, GX-NN10, GX-NN8, GX-YL7 and reference vaccine strain 4/91 were classified into the 4/91-type, but the latter five isolates occupied another offshoot. GX-YL6 and reference strains LDT3, Partridge/GD/S14/2003 and Korea strain KM91 were grouped as the tl/CH/LDT3/03-type. Amazingly, GX-G and GX-XD isolated in 1988 were grouped with Taiwan reference strains TW2296/95, TW2575/98 as Taiwan-type. Isolates GX-NN1 and GX-NN2 showed a close relationship with commonly used vaccine strains H120, H52, Ma5, M41 and other China vaccine strains W93, H94, D41, IBN, HK and grouped as Mass-type. GX-C, isolated in 1985, showed considerable low homology with the above five genotypes and belonged to a separate group.

The phylogenetic trees of M and N genes showed that the 23 isolates were segregated into 4 distinct groups, which exhibited considerably different topology than that of the S1 gene (Figure 1b,c). In the phylogenetic trees of the M gene, 12, 2, 3 and 6 isolates were designated as CK/CH/LSC/99I-type, LX4-type, Mass-type and BJ-type respectively. In the phylogenetic trees of the N gene, 9, 4, 9 and 1 isolates were designated as LX4-type, new-type, CK/CH/LSC/99I-type and Mass-type respectively.

### 2.3. Positive Selection on the S1, M and N Proteins of IBVs

The results of the codon-based tests of positive selection (Z-test, MEGA5) for analyzing the numbers of non-synonymous and synonymous substitutions per site (dN/dS ratio) on the S1, M and N proteins were displayed as supplementary material (Supplementary Figure S1). No significant evidence for positive selection of S1 protein of Taiwan-type and Mass-type groups was observed (P > 0.05). However, 33.3% (5/15) of the pairwise comparisons of 4/91-type strains and 75.8 % (50/66) of the pairwise comparisons of CK/CH/LSC/99I-type strains showed positive selection (P < 0.05) on S1 protein. Results of the Z-test of M protein revealed that 86.4% (57/66) of the pairwise comparisons of CK/CH/LSC/99I-type strains and 73.3% (11/15) of the pairwise comparisons of BJ-type strains were under positive selection (P < 0.05). 93.3% (42/45) of the pairwise comparisons of CK/CH/LSC/99I-type strains and 77.8% (35/45) of the pairwise comparisons of LX4-type strains were subjected to positive selection (P < 0.05) on N protein. Positive selections were identified between any pair of groups in the corresponding genes. 

### 2.4. Analysis of Entropy of Amino Acid Sequences

**Figure 2:**
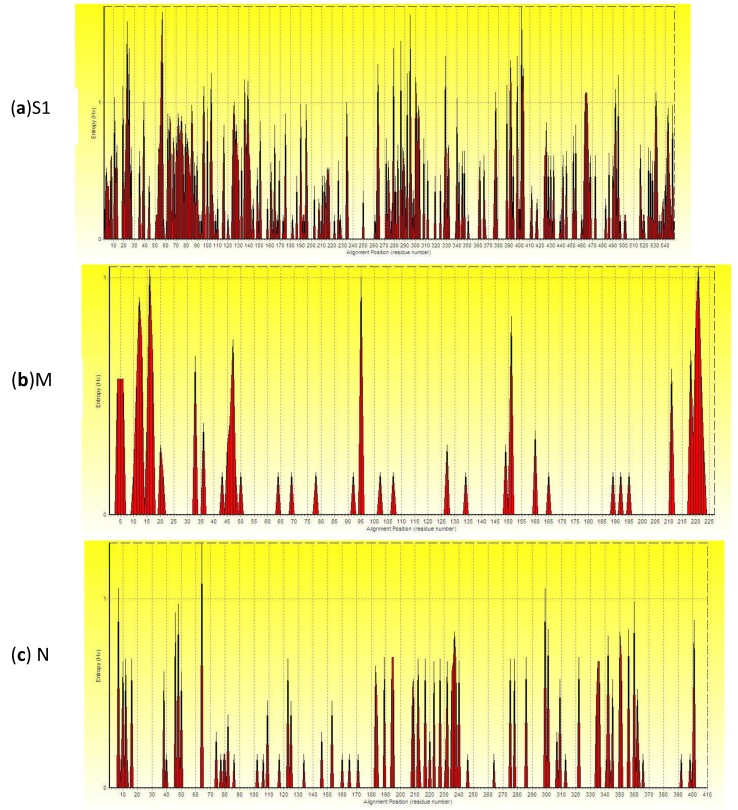
Entropy plot of amino acid of (**a**) S1, (**b**) M and (**c**) N protein gene of IBV. X-axis gives the amino acid sites of S1, M and N gene; y-axis gives the entropy of each amino acid site.

The result of analysis of entropy of S1, M and N genes on amino acid sequences was shown in Figure 2. The higher the peak is, the greater the entropy is, indicating the higher variation frequency of amino acid sites. Numerous high entropy amino acid sites were distributed throughout the entire S1 gene; only a few high entropy amino acid sites were scattered within the M gene. The number of high entropy amino acid sites within the N gene is less than that of the S1 gene but more than that of the M gene (Supplementary material). An entropy value bigger than 0.4 indicated the corresponding amino acid site was not conserved. The percentages of entropy bigger than 0.4 in amino acids sequences of S1, M and N gene were 29.87% (164/549), 6.61% (15/227) and 11.46% (47/410), respectively. The descending average entropy order were S1 (0.2651) > N (0.0953) > M (0.0831). Therefore, the S1 gene amino acid sequences had the largest variation; the M gene had less variation than the N gene.

### 2.5. Analysis of Recombinants

**Figure 3:**
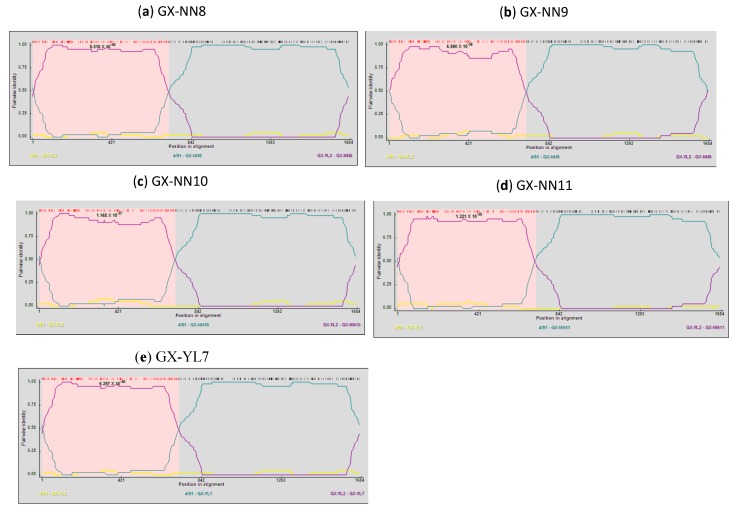
Recombination Detection Program (RDP) screenshots displaying the possible recombination events on the isolates (**a**) GX-NN8, (**b**) GX-NN9, (**c**) GX-NN10, (**d**) GX-NN11 and (**e**) GX-YL7. Each panel displays the pairwise identities among the possible mosaic and its putative parents. Pairwise identity refers to the average pairwise sequence identity within a 40-nt sliding window moved one nucleotide at a time along the alignment of the three sequences. The pink area demarcates the potential recombination regions.

Recombinant events were detected in the S1 gene of isolates GX-NN8, GX-NN9, GX-NN10, GX-NN11 and GX-YL7 by all recombination detection methods implemented in the RDP4.14 software. These five isolates were found to be recombinants between the vaccine strain 4/91 and the CK/CH/LSC/99I-Type field strain GX-YL2 (Figure 3) with very high significance of RDP (8.717 × 10^−38^), GENECONV (8.068 × 10^−32^), BootScan (3.608 × 10^−37^), MaxChi (1.894 × 10^−25^), Chimaera (1.377 × 10^−25^), SiScan (8.414 × 10^−31^) and 3Seq (8.416 × 10^−71^). Their crossover regions were observed at nucleotide position 7–677 or 7–678 (7–718 in alignment). Both the N-terminal and the C-terminal of the S1 gene sequence of these five isolates showed high similarity with CK/CH/LSC/99I-type isolate GX-YL2 (98.5–99.2%) and 4/91 strain (99.3–99.5%), respectively. Significantly discrepant topologies of phylogenetic trees (Supplementary Figure S2) and the results of Similarity plot and BootScan analyses (data not shown) supported further recombinant events of them.

## 3. Discussion

One of the major problems caused by IBV in the field is the frequent emergence of new variants. IBV strains within a geographic region are unique and distinct [19]. Outbreaks of IB still occurred in Guangxi [15,17] although vaccines have been applied and the molecular epidemiology information available was limited. Hence, we investigated the genetic characteristics of S1, M and N genes of IBVs circulating in this region. This is the first report on the analysis of entropy of amino acid sequence and the positive selection of S1, M and N genes of IBVs. 

Some investigators reported the genetic typing based on HVR I of the S1 gene is representative of the grouping based on the whole S1 gene [8,24], but another study disagreed with these findings [25]. The present results and our previous report from HVR I [15] also indicated that genotypes based on HVR I are not representative of that based on the whole S1 gene. The reason was that mutations in non-HVR I of the S1 gene were also detected [25]. In addition, In addition, our results showed that genotypes based on S1 gene exhibited considerably difference from M and N genes. The discordance of topology in the S1-based tree and other gene-based trees were also described by other investigators [26,27].

The co-circulation of multiple IBV types and the ongoing emergence of IBV variants are the epidemiological challenges in China. Nine genotypes including LX4-type, CK/CH/LSC/99I-type, tl/CH/LDT3/03-type, CK/CH/LDL/97I-type, BJ-type, CK/CH/LHLJ/95I-type, Mass-type, 4/91-type, and N1/62 associated circulated in China and LX4-type was the dominant genotype [20]. Recently, the Taiwan II-type was firstly reported in China [22]. In our study, CK/CH/LSC/99I-type, tl/CH/LDT3/03-type, 4/91-type, Taiwan-type and Mass-type were identified, which suggested that multiple genotypes of IBVs were co-circulating in Guangxi province. Eleven out of 23 isolates sharing 76.8–77.9% S1 gene amino acid sequence similarity with the vaccine strain H120 belonged to CK/CH/LSC/99I-type. However, the prevalent genotype in this region was CK/CH/LSC/99I-type not the LX4-type, which was different from other reports that LX4-type was the dominant genotype in China [20,22], indicating IBV strains within this region are unique and distinct. 

IBV has been diagnosed in China since the early 1980s [20]. Surprisingly, the GX-G and GX-XD isolated in 1988 showed closed relationship with Taiwan II–type strain TW2296/95 and no Taiwan-type strain occurred in recent year in our study. Recently other investigators reported that Taiwan II–type strains of IBV occurred in mainland China [22]. Whether the Taiwan-type IBVs entered China by long distances migration of wild birds or importing of poultry products or improper use of vaccines？It is unclear. Identification of Tai-wan and China-like recombinant IBVs in Taiwan was reported [28]. So it is very important to monitor the vaccine, birds and poultry products in China.

Natural selection generally causes a reduction in deleterious mutations while promoting advantageous mutations. A gene which undergoes positive selection promoted by natural selection usually has highly important functions [29]. Some investigators reported positive selection wasn’t detected in the spike protein of IBVs although they differed markedly in the sequence of the spike protein [26,30]. However, other investigators showed different results. Positive selection was detected in the spike protein of IBV California-type viruses, for which no vaccine exists but was not detected in Massachusetts- and Connecticut-types where attenuated live vaccines are routinely used [11]. Another report showed that positive selection was found in the S1 protein of variants isolated from layer-type birds but was not found in variants isolated from broilers, even though a high number of mutations was significantly associated with broiler-type chickens [25]. Positively selected sites in the nucleocapsid protein of the Taiwan IBV and their effects on RNA-binding activity were reported recently [31]. Previous reports on SARS-CoV indicated that positive selection on S protein was changeable in different epidemic groups and positive selection on replicase of SARS-CoV was detected only in human patients, not in any proteins of bat SARS-like-CoV [29]. We found positive selection was observed on S1 protein of 4/91-type and CK/CH/LSC/99I-type strains, M protein of CK/CH/LSC/99I-type and BJ-type strains, N protein of CK/CH/LSC/99I-type and LX4-type strains. Thus, not only S1 protein but also M and N proteins experienced positive selection during the IBV epidemics. The variation of positive selection of S, M and N proteins among different groups may explain why these field variants escape immune pressure and may provide valuable evidence that these three structural proteins may be critical for virus evolution. It is the first time to analyze the positive selection of S1, M and N genes of IBVs.

The entropy is one useful quantification of diversity in a single position of amino acid sequences [32]. High scoring amino acid positions may correlate with structurally or functionally important residues [33]. The greater the entropy is, the higher variation frequency of amino acid sites is. An entropy value bigger than 0.4 indicated the corresponding amino acid site was not conserved [34]. A Shannon entropy analysis of immunoglobulin and T cell receptor revealed that the T cell receptor is significantly more diverse than immunoglobulin-suggesting T cell receptor has new complementarity determining regions, which represent a larger antigen combining site, additional combining sites, or an evolutionary strategy to avoid inappropriate interaction with other molecules [35]. A recent study used the Shannon entropy and relative entropy to measure the diversity of amino acid site of H3 HA between the 1992–1993 season and the 2009–2010 season and showed that the rate of evolution increases with the virus diversity in the current season and the Shannon entropy of the sequence in the current season predicts relative entropy between sequences in the current season and those in the next season [32]. According to our results, the average entropy of amino acid sequences, the percentages of entropy bigger than 0.4 and the number of amino acid sites with high entropy of S1 gene are biggest, and those of N gene were bigger than M gene. Thus, these observations revealed that amino acid sequences of S1 gene had the largest variation; the M gene had less variation than the N gene. To our knowledge, it is the first time to analyze the entropy of S1, M and N gene amino acid sequences. The variation of amino acids will have an important effect on the biological function and evolution of viruses. Hence, observing the biological function of the amino acid residues with higher entropy and identifying the positively selected sites among IBVs will be further studied. 

Recombination is involved in the emergence and evolution of IBV or can even directly lead to the emergence of new coronaviruses and related diseases [36] Recombination can occur between field isolates or between field and vaccine viruses [36,37,38]. In our study, convincing evidence showed five S1-gene recombinants GX-NN8, GX-NN9, GX-NN10, GX-NN11 and GX-YL7, with their putative parental strains of vaccine strain 4/91 and CK/CH/LSC/99I-Type field strain GX-YL2, and their crossover regions were at nucleotide position 7–677 or 7–678. A recently report showed a recombinant (ck/CH/LZJ/111113 strain) came from a Chinese field isolate (ck/CH/LDL/091022 strain, LX4-type) and a 4/91-like strain, with switches at 3 sites, namely upstream of S, the N gene and the 3' UTR [39]. Besides the Mass-type vaccine, 4/91-type live vaccines are also commonly used in China including during the breeding period [39], even without official authorization. Our finding provides another evidence that 4/91 vaccine strains are contributing to the emergence of variants in the field in China. Therefore, it is necessary to strengthen the vaccine licensing system before introduction of exotic IBV strains. We should continued 49/1-type recombinants surveillance in China. The pathogenicity of 4/91-derived recombinants should be assessed in further studies.

## 4. Experimental Section

### 4.1. Virus Isolation and Propagation

Twenty-three IBV strains, isolated as previously described [15] were analyzed in the present study. The IBV field isolates were propagated in 9 to 11-day-old specific pathogen free embryonated chicken eggs via the allantoic cavity route. Allantoic fluids were harvested at 48 h post-inoculation, frozen, and stored at −70 °C until used.

### 4.2. Primers for S1, M and N genes Amplification

For each IBV strain, the entire S1, M and N genes were amplified. The S1 primers were designed according to the previous report and the anticipated amplification segment is about 1760 bp encompassing the entire S1 gene including the protease cleavage motif [40]. The M gene sense primer was:5'-CGAGTTTCCTAAGAACGGTTGGAA-3', and the anti-sense primer was: 5'-CCCCTCTCTACACGCACACATTTAT-3'. The N gene sense primer was: 5'-CCATGGCAAGCGGTAAAGCAR-3', and the anti-sense primer was: 5'-CCACTCAAAGTTCATTCTCTCC-3'. The anticipated amplification segments for M and N genes are 750 bp and 1236 bp respectively. 

### 4.3. RNA Extraction and Amplification of S1, N and M Genes

Viral RNA was extracted from the infectious allantic fluid by the Trizol reagents (Invitrogen, USA) according to the manufacturer’s instruction. The first cDNA strand was synthesized in 25 µL mixture consisting of 9 µL of RNA extract, 1µL of 50 µM/µL Random 9 mers, 5µL of 5 ×reverse transcriptase first strand buffer, 1 µL of 40 U/µL RNase inhibitor (TaKaRa, Japan), 1 µL of 200 U/µL AMV reverse transcriptase (TaKaRa, Japan) and 8µL of 2.5mmol/L dNTPmix (TaKaRa, Japan). The mixture was incubated at 42 °C for 1 h, and then inactivated at 99 °C for 5 min. For the following PCR assays, a total of 25 µL reaction mixture consisted of 2 µL of the cDNA, 2.5 µL of 10× PCR buffer, 2 µL of 2.5 mmol/L dNTPmix (TaKaRa, Japan), 1 µL of 25 µmol/L of each of the two primers and 0.25 µL of 5 U/µL Taq DNA polymerase (TaKaRa, Japan). The PCR conditions for the S1 gene amplification were 94 °C for 6 min, 35 cycles of 94 °C for 45 s, 55 °C for 45 s, and 72 °C for 2 min, followed by 72 °C for 10 min; that for the M gene were 94 °C for 5 min, 35 cycles of 94 °C for 1min, 50 °C for 1 min, and 72 °C for 1 min, followed by 72 °C for 10 min; and that for the N gene were 94 °C for 5 min, 35 cycles of 94 °C for 1 min, 50 °C for 1 min, and 72 °C for 2 min, followed by 72 °C for 10 min. The PCR products were analyzed on 1.0% agarose-gel electrophoresis.

### 4.4. Gene Sequencing, Alignments and Phylogenetic Analysis

The PCR products were purified, cloned and then sequenced by Sangon Bio-company (Shanghai, China). For each gene, three independent clones were selected randomly and sequenced twice from both directions. The open reading frames of S1, M and N gene were determined using the DNAstar version (DNAStar, Madison, WI). The nucleotide sequences of S1, M and N genes have been submitted to GenBank database and assigned accession numbers (Supplementary Table S2). The nucleotide and the deduced amino acid sequences alignments were generated using the ClustalW Multiple Alignment method of BioEdit version 7.0.9.0 and compared with those of 42 reference IBV strains retrieved from the GenBank database with the accession numbers listed in in supplementary material (Supplementary Table S3). Phylogenetic trees were constructed based on the amino acid sequences of S1, M and N genes with the Neighbor-joining method (Jones-Taylor-Thornton (JTT) model) and Maximum-likelihood method (JTT model) using MEGA 5.05 version. The bootstrap values were determined from 1000 replicates of the original data. 

### 4.5. Analysis of Entropy of Amino Acid Sequences and Positive Selection

The entropy is one useful quantification of diversity in a single position of amino acid sequences. A large entropy means the amino acid in the given position is prone to be substituted. In order to understand the variation degree of S1, M and N genes, the entropy of aligned amino acid sequences within these genes of the isolates was calculated by BioEdit version 7.0.9.0. In addition, codon-based tests of positive selection (Z-test, MEGA5) were used to estimate the numbers of non-synonymous and synonymous substitutions per site (dN/dS ratio) within the S1, M and N proteins in order to understand whether these proteins are submit to positive selection.4.6. Computational Recombination Analysis.

Aligned nucleotide sequences of S1, M and N genes were analyzed with the Recombination Detection Program (RDP4, Version 4.14) to detect potential within-gene recombination events. The window size was adjusted to 40 bp from the default setting 30 bp because IBV has a high mutation rate, which can mask recombination signals. The highest acceptable P value was 0.05 and the detection of recombination events was applied between sequences sharing 0 and 100% identity. Seven algorithms in RDP 4.14, including RDP, GENECONV, BootScan, MaxChi, Chimaera, SiScan and 3Seq were used to confirm the recombination events. Two phylogenetic trees, which were constructed from the portion of the alignment between the inferred breakpoints and the remainder of the alignment were made and compared to assess recombination events further. Recombination events and recombination breakpoints were further confirmed by Similarity plot and BootScan analyses using the SimPlot program (version 3.5.1.).

## 5. Conclusions

In conclusion, multiple IBV genotypes co-circulated; genetic diversity and positive selections existed in S1, M and N genes; 4/91 vaccine recombinants emerged in China. Our results show that field IBVs in China are continuing to evolve and vaccine strains may have an important role in the appearance of new IBV strains via recombination. In addition, the present study indicates that IBV evolution is driven by both generations of genetic diversity and selection.
